# Isoxazole Derivatives as Regulators of Immune Functions

**DOI:** 10.3390/molecules23102724

**Published:** 2018-10-22

**Authors:** Michał Zimecki, Urszula Bąchor, Marcin Mączyński

**Affiliations:** 1 Laboratory of Immunobiology, Institute of Immunology and Experimental Therapy, Polish Academy of Sciences, Weigla 12, 53-114 Wroclaw, Poland; zimecki@iitd.pan.wroc.pl; 2 Department of Organic Chemistry, Faculty of Pharmacy, Wroclaw Medical University, Borowska 211a, 50-556 Wroclaw, Poland; urszula.bachor@umed.wroc.pl

**Keywords:** isoxazoles, anti-inflammatory, immune suppression, immune stimulation

## Abstract

In this review, we present reports on the immunoregulatory properties of isoxazole derivatives classified into several categories, such as immunosuppressive, anti-inflammatory, immunoregulatory, and immunostimulatory compounds. The compounds were tested in various models using resident cells from rodents and humans, cell lines, and experimental animal disease models corresponding to human clinical situations. Beneficial features of the described isoxazole derivatives include low toxicity and good bioactivity at low doses. In a majority of studies, the activities of investigated compounds were comparable or even higher than registered reference drugs. Whenever possible, a plausible mechanism of action of the investigated compounds and their potential therapeutic utility were proposed. Among the described compounds, particular attention was paid to the class of immune stimulators with a potential application in chemotherapy patients.

## 1. Introduction

Compounds containing the isoxazole ring [1] serve as an important source of valuable drugs that are designed to treat infections and diseases of different etiologies. Syntheses, classification, mechanisms of action, and the therapeutic application of registered isoxazole derivatives, as well as these under preclinical investigations, were recently in detail described in several reviews [2,3,4,5]. Therefore, we wished to focus in this short review only on data regarding the immunoregulatory properties of compounds containing the isoxazole ring. Although a majority of isoxazole derivatives under interest display immunosuppressive activities, including anti-inflammatory ones, less is known about immunostimulatory compounds of potential therapeutic utility. Such a group of compounds, besides immunosuppressive molecules, was also synthesized, and their potential therapeutic usefulness evaluated by our research team. In this review, the results regarding immunoregulatory actions of selected isoxazole derivatives in various immunological settings in vitro and in vivo human and rodent models are described (Table 1). In some investigations, the description of the immunoregulatory properties is accompanied with the suggested mechanism of action, and potential therapeutic applications are proposed.

## 2. Results and Discussion

### 2.1. Immune Suppression

Leflunomide and its active metabolite [6] are the most well-recognized immunosuppressive drugs, and they have found therapeutic applications if used alone or in combination with other drugs in rheumatoid arthritis [40] or transplantation [41]. As a prime representative of the isoxazole family, the drug is widely used as a reference drug to compare the potency of other investigated isoxazoles. Another example of a widely used isoxazole drug is Parecoxib, a pain reliever that acts as cyclooxygenase 2 (COX-2) inhibitor [7]. Its safety has been a subject of many multicenter trials, as summarized in a recent article [42], which could not definitely confirm the lack of its side effects reported earlier [43].

In investigations with rodents, a number of isoxazole derivatives was evaluated for their in vitro and in vivo immunosuppressive activities. In a study on the derivatives of isoxazole[4,5-*d*]pyrimidine [8], two compounds, **8g** and **10f**, inhibited the humoral immune response to sheep erythrocytes (SRBC) in vivo, but only one of them suppressed (**10f**) the delayed-type hypersensitivity response (DTH), showing that closely related compounds may differentially affect these two major types of immune response.

Suppressive properties of 5-amino-3-methylisoxazole-4-carboxylic acid amides and ureilenes [44], and 5-aminomethinimino-3-methyl-4-isoxazolecarboxylic acid phenylamides [9] on the humoral and cellular immune response in mice were subsequently found. One of the compounds, 5-(2-hydroxyethyl)piperazinomethinimino-3-methyl-4-isoxazolecarboxylic acid 4-(4-ethoxyphenyl)-amide (**4d**) [9] exhibited an exceptionally strong level of activity, comparable to cyclosporine. In turn, a study on the immunosuppressive activities of 5-substituted 3-methylisoxazole[5,4-*d*]-1,2,3-triazin-4-one derivatives [45], was supplemented with quantum-chemical calculations using AM1, a semi-empirical method for geometry optimization, the estimation of descriptor values, and the localization of the HOMO and LUMO orbital. The structure/activity investigations were extended by the application of the B3LYP hybrid exchange-correlation energy functional and the 6-31G(d, p) basis set. [46] The Polarizable Continuum (SCRF/PCM) solvent model was also taken into account, in order to show the solvent influence on the electron density, and the electrostatic potential around the exemplary molecules. Correlations between the molecular structure and its biological properties were subsequently found by using a stepwise selection of scales for the multiple linear regression (MLR).

The studies on an isoxazole[5,4-*e*]triazepine derivative (compound **RM33**) led us to a demonstration of the very potent immunosuppressive activities of the compound in mouse models [10,47]. The compound effectively suppressed both the humoral response to SRBC, and the cellular response to ovalbumin (OVA) when administered intraperitoneally or per os. The compound could also act locally by the inhibition of foot pad edema when admixed with Freund’s complete adjuvant. In addition, serum tumor necrosis factor alpha (TNF α) and interleukin 6 (IL-6) levels, induced by administration of lipopolysaccharide (LPS) were reduced, but not an inducible interleukin 10 (IL-10) concentration in splenocyte cultures. **RM33** inhibited also foot pad edema that was induced by carrageenan in Wistar rats [48], accompanied by lower serum TNF α levels. The inhibition of the foot pad edema was correlated with adequate histological changes, such as a lesser level of damage to mast cells, and smaller macrophage infiltration. Very recent (unpublished data) indicate that the immunosuppressive action of **RM33** may consist of its apoptotic action on thymocytes and splenocytes, associated with strong increases of caspase 9, p53, and NFκB1 and NFκB2 expression.

In another investigation of our group, a series of 5-amino-3-methylisoxazole[5,4-*d*]pyrimidin-4-one derivatives were obtained by the reaction of *N*′-substituted derivatives of 5-amino-3-methyl-1,2-oxazole-4-carbohydrazide with ethyl orthoformate [11]. The compounds differentially inhibited in vivo cellular and humoral immune responses in mice and pokeweed mitogen (PWM)-induced polyclonal antibody production in a culture of human peripheral blood mononuclear cells (PBMC). The derivatives displayed differential inhibitory activities in these models, depending on the character and location of the substituted groups. Also, a significant suppression of the in vivo humoral immune response to SRBC in mice was achieved upon low doses of 5-amino-3-methyl-4-isoxazolecarboxylic acid semicarbazides and thiosemicarbazides [12]. Other studies on new lead structures of the isoxazole system [13] revealed their strong immunosuppressive activities in the humoral immune response, the carrageenan model, and the proliferative response of lymphocytes. Density Functional Theory (DFT) was employed to shed light on the molecular properties of the investigated compounds. The geometrical parameters of the studied molecular structures were fully optimized at the B3LYP/6-311G (d, p) level. The atomic charge distribution, derived on the basis of the Mulliken population analysis, was correlated with the immunological activity of the compounds. The obtained relationships revealed that the isoxazole ring played an important role in the observed immunological activities.

In a study on 5-amino-3-methyl-4 isoxazolecarboxylic acid benzylamides [14] a selected **MO5** compound (5-amino-3-methyl-*N*-[(4-methylphenyl)methyl]-1,2-oxazole-4-carboxamide) inhibited the humoral immune response in vitro and stimulated the inductive phase of DTH in vivo, although it suppressed the eliciting phase of that response and the foot pad carrageenan reaction. Its action on phytohemagglutinin A (PHA)-induced PBMC proliferation, and TNF α production was also inhibitory. In a parallel study [15], effects of 5-amino-3-methyl-1,2-oxazole-4-carbohydrazide on selective gene expression in Caco-2 cells were studied, together with Leflunomide (5-methyl-*N*-[4-(trifluoromethyl)phenyl]-4-isoxazolecarboxamide), as a reference drug. It appeared that both compounds similarly regulated selected genes i.e., the upregulation of fractalkine (CX3CL1) and IL-17F, and the downregulation of IL-10 and toll-like receptor 4 (TLR4). Nevertheless, the expression of 12 genes was differently regulated by these compounds: interleukins (IL) IL-1B, IL-6, and a chemokine CCL22 were upregulated by the compound, but significantly suppressed by Leflunomide. IL-2 and IL-27 in turn were upregulated by Leflunomide and suppressed by the compound. The authors concluded that the studied compound has a potential for further modifications as an immunosuppressive agent, with applications that are different from that of Leflunomide.

In a new series of *N*′-substituted derivatives of 5-amino-*N*,3-dimethyl-1,2-oxazole-4-carbohydrazide [16], a selected 5-amino-*N*′-[2-(2,4-dihydroxyphenyl)ethylidene]-*N*,3-dimethyl-1,2-oxazole-4-carbohydrazide (**MM3** compound) lowered the PHA-induced proliferation of PBMC and LPS-induced TNF α production in human blood cell culture. In the model of Jurkat cells, **MM3** elicited strong increases in the expression of caspases, Fas, and NF-κB1, indicating that a proapoptotic action may account for its immunosuppressive action in the studied models.

### 2.2. Inhibition of Inflammation

Izoxazole derivatives exhibiting anti-inflammatory properties constitute a major group from this class of potential therapeutics. In an early study [21] the authors demonstrated strong anti-inflammatory and antibacterial activity of *p*-etoxyphenylamid and *p*-chlorophenylamid of 5-benzoylamino-3-methyl-4-isoxazolecarboxylic acid. The most potent activity of these compounds were attributed to the benzoyl group in position 5 of the isoxazole ring.

**HWA-486** (5-methyl-*N*-[4-(trifluoromethyl)phenyl]-1,2-oxazole-4-carboxamide), together with cyclophosphamide, a reference drug, was investigated in the model of adjuvant arthritis in Lewis rats [17]. The compound prevented the development of the disease and suppressed the mitogen-elicited proliferation of lymphocytes derived from adjuvant arthritis rats. In contrast to cyclophosphamide, the compound did not affect the proliferation of lymphocytes from healthy rats.

Leflunomide and its active metabolite A771726 [49] suppressed the immune response by means of various mechanisms [50,51]. In a model of J774.2 macrophages treated with LPS, both agents were shown to inhibit PGE2 accumulation in cell cultures. Further, in a whole blood human cell culture stimulated with calcium ionophore, Leflunomide inhibited both COX-1 and COX-2 activity [50]. In vivo effects of the compound were restricted by experimental substrate supply, and probably by plasma binding. Other important actions of A771726 include suppression of proinflammatory cytokine production, such as TNF α and IL-1 [51].

In an extensive study on in vitro, ex vivo, and in vivo immunopharmacological effects of **VGX-1027** (*S*,*R*)-(3-phenyl-4,5-dihydro-1,2-oxazol-5-yl)acetic acid [22], the investigators evaluated the potential efficacy of the compound in the amelioration of acute and chronic immunoinflammatory diseases, based on its ability to inhibit LPS-induced cell signaling pathways. A lack of toxicity predisposes the compound for further preclinical investigations.

A potential utility of new fatty acid amine hydrolase (FAAH) inhibitors based on 3-carboxamido-5-aryl-isoxazole scaffolds in protection against experimental colitis was recently also proposed [23].

A search for novel anticancer drug via inhibition of LOX and COX enzymes constitutes an important strategy, since the overproduction of leukotrienes and prostaglandins contributes to tumor growth by inducing formation of new blood vessels that support tumor cell growth. It was demonstrated [24] that one of the synthesized compounds, 5-(3-methylthiophen-2-yl)-3-(3,4,5-trimethoxyphenyl)-1,2-oxazole (**2b**) exhibited significant inhibitory activity toward lipooxygenase (LOX) and COX-2. 4,5-diarylisoxazol-3-carboxylic acids could also serve as leukotriene synthesis inhibitors [25], and thus they are potential anti-inflammatory drugs.

Macrophage migration inhibitory factor (MIF) is an inflammatory cytokine that is associated with inflammatory disorders, as only cytokine is resistant to steroid action. Airway hyper-responsiveness is also relatively resistant to steroid therapy. A MIF antagonist (*S*,*R*)-methyl [3-(4-oxocyclohexa-2,5-dien-1-ylidene)-1,2-oxazolidin-5-yl]acetate (**ISO-1**) [26] proved to be efficient in the suppression of lung inflammation in ozone-exposed mice, in contrast to Dexamethasone. MIF is also implicated in development of autoimmunity, so that the therapeutic efficacy of **ISO-1** was also evaluated in a model of glomerulonephritis in lupus-prone NZB/NZW F1 and MLR/lpr mice [52]. The results showed that **ISO-1**, like anti-MIF, inhibited the interaction between MIF and its receptor, and reduced the functional and histological parameters of glomerulonephritis, leukocyte recruitment, proinflammatory cytokine and chemokine expression. These data support the therapeutic value of the compound by the downregulation of MIF-dependent pathways of tissue damage in systemic lupus erythematosus. For the prevention of another type of autoimmunity, (*S*,*R*)-(3-phenyl-4,5-dihydro-1,2-oxazol-5-yl)acetic acid (**VGX-1027**) was tested in a model of NOD mice, with spontaneous or accelerated forms of diabetes induced either by injection of cyclophosphamide or by transfer of spleen cells from acutely diabetic syngeneic donors, representing a preclinical model of human type 1 diabetes mellitus [53]. The compound significantly diminished the cumulative incidence of diabetes and insulitis in this model. The animals receiving VGX-1027 exhibited reduced production of the proinflammatory mediators, such as TNF α, IL-1β, and MIF as well as inducible nitric-oxide synthase-mediated nitric oxide generation in both pancreatic islets and peripheral organs. The authors recommended **VGX-1027** for further investigations as a prime candidate to treat this type of the autoimmune disease.

To suppress pulmonary inflammation, a novel glucocorticoids series of (GCs), 6α,9α-di-fluoro 3-substituted C-16,17-isoxazolines was designed [27]. A selected Cpd #15 compound inhibited LPS-induced nitric oxide release in Raw264.7 mouse macrophages, and TNF α-induced IL-8 production in human airway smooth muscle cells with a potency higher than that of Dexamethasone. The authors concluded that the compound displayed a pharmacokinetic and pharmacodynamic profile that was suitable for topical treatment of conditions that are associated with lung inflammatory reactions.

A series of new isoxazole derivatives of expected immunosuppressive activities was synthesized [29]. Following in vitro screening in the human cell models, the activity of a most active **MZO-2** compound (ethyl *N*-(4-{[(2,4-dimethoxyphenyl)methyl]carbamoyl}-3-methyl-1,2-oxazol-5-yl)ethanimidate) in mouse in vivo models was evaluated. The compound, administered intraperitoneally, inhibited carrageenan-induced footpad edema, and the contact sensitivity in mice to oxazolone when applied in ointment, with a potency that was comparable to tacrolimus (Protopic^®^). The compound inhibited also expression of caspases 3, 8, and 9 in Jurkat cells.

Several new 3-methylisoxazol-5(4*H*)-one/2-hydroxy/mercapto-6-methylpyrimidin-4(5*H*)-one/3-methyl-1-substituted-1*H*-pyrazol-5(4*H*)-one substituted benzimidazole derivatives [30], were synthesized. A most potent representative of this series, 4-(2-(4-(1*H*-benzimidazol-2-yl)phenyl)hydrazono)-1-(4-chlorophenyl)-3-methyl-1*H*-pyrazol-5(4*H*)-one demonstrated significant analgesic activity, protection in carrageenan-induced footpad edema, efficient antibacterial, and moderate antiviral activities. Among the triazines, novel series [28] were synthesized, including isoxazoles with COX-2 inhibitory activity. To evaluate their anti-inflammatory activities, the compounds were investigated by the paw thickness inhibition assay. The most potent compound in the group of isoxazole derivatives that reduced the carrageenan-induced mice paw edema in comparison to Celecoxib, was compound **7a** (*N*-(4-(isoxazol-5-yl)phenyl)-4,6-di(piperidin-1-yl)-1,3,5-triazin-2-amine) (51% inhibition of paw edema). Most of the compounds were even more potent than the reference drugs, such as: Celecoxib, Diclofenac, and Indomethacin. Analgesic and anti-inflammatory properties were also found when investigating isoxazole–mercaptobenzimidazole hybrids [31].

Indolyl–isoxazolidines are another category of compounds with potent anti-inflammatory-analgesic activities [32]. A selected compound, **9a**, significantly inhibited LPS-induced TNF α and IL-6 production in macrophage THP-1 cells. The compound also caused analgesia and showed anti-inflammatory effects in the carrageenan test, with a potency that was comparable to that of indomethacin. Compound **9a** was also effective in the protection of LPS-induced death in mice, indicating its potential usefulness as an analgesic/anti-inflammatory drug.

### 2.3. Immunoregulation

Following demonstration of the suppressive effects of **HWA-486** (Leflunomide) compound in adjuvant-induced arthritis in rats using cyclophosphamide, prednisolone, and cyclosporine A as reference drugs [17], the studies were extended by using several in vivo and ex vivo methods that were aimed at the elucidation of differences between the mechanisms of the actions of **HWA-486** and of reference immunosuppressors. The authors came to a conclusion [17] that the compound has inhibitory activity on T-cell-dependent B-cell immune responses, yet it does not affect T-cell-independent B-cells; thus T-cell responsiveness may not be affected. Therefore, these findings may explain the disease-modifying activity of **HWA-486** in adjuvant-induced arthritic rats.

Immunoregulatory actions were described in a series of reports on the derivatives of phenylamides of 5-amino-3-methyl-4-isoxazolecarboxylic acid [44]. The activities of the compounds in the proliferative response of PBMC to PHA, and in LPS-induced cytokine production in the PBMC cultures were differential. The stimulatory or inhibitory effects depended strongly on the origin and location of substituents in the phenyl ring, as supported by QSAR (Quantitative Structure–Activity Relationship) studies. Similar conclusions could be drawn from in vivo and in vitro investigations on 4-imino derivatives of the 5-amino-3-methyl-1,2-oxazole-4-carbohydrazide and 5-amino-3-methylisoxazole[5,4-*d*]-6,7-dihydropyrimidine in mouse models [54].

Immunoregulatory properties were also observed with derivatives of 5-amino-3-methyl-1,2-oxazole-4-carbohydrazide [18]. The compound exhibited modulatory effects on T-cell subsets and levels of B-cells in lymphoid organs, and enhanced anti-SRBC antibody production in mice. The authors suggest its potential use in the treatment of autoimmune diseases, infections, or as an adjuvant for boosting the efficacy of vaccines.

The immunoregulatory properties of 2-(5-amino-3-methyl-1,2-oxazole-4-carbonyl)-*N*-(4-chlorophenyl)hydrazine-1-carbothioamide (**06K** compound) in mouse in vivo models were also investigated [19]. Interestingly, the actions of the compounds were differential, depending on a model used. The compound stimulated antibody production against SRBC, as measured by the numbers of antibody-forming cells, but the cellular immune response to OVA was decreased. Further, the compound significantly diminished carrageenan-induced foot pad edema. Phenotypic studies revealed that the immunoregulatory properties of the compound involved the mobilization of lymphopoiesis, and the generation of regulatory T cells. In another report with the application of in vitro models [20] 2-(5-amino-3-methyl-1,2-oxazole-4-carbonyl)-*N*-phenylhydrazine-1-carbothioamide (**01K**), and 2-(5-amino-3-methyl-1,2-oxazole-4-carbonyl)-*N*-(4-chlorophenyl)hydrazine-1-carbothioamide (**06K**) exhibited regulatory activity in the proliferation tests using cells from the thymus, spleen, and lymph nodes, as well as in the effects of the compounds on IL-1β, and the production of TNF-α in peritoneal cell cultures. These actions were associated with the different influence of the compounds on signaling protein expression in immature T cell Jurkat cell lines. Immunosuppressive isoxazole Leflunomide and a stimulatory **RM-11** compound (1,7-dimethyl-8-oxo-1,2H-isoxazole[5,4-*e*]triazepine) were applied as reference drugs.

A study on the immunoregulatory properties of the selected 5-amino-3-methyl-4-isoxazolecarboxylic acid benzylamides [14], and its selected derivative **MO5** (5-amino-3-methyl-*N*-[(4-methylphenyl)methyl]-1,2-oxazole-4-carboxamide), showed that the compounds inhibited the humoral immune response in vitro, stimulated the inductive phase of DTH in vivo, although it inhibited the eliciting phase of that response. The compound also inhibited the carrageenan skin reaction in mice, and inhibited LPS-induced TNF α production in human whole blood culture, as well as PHA-induced proliferation of PMBC.

### 2.4. Immunostimulation

Immunostimulatory actions of therapeutics are equally important as immunosuppressive ones, and they are invaluable in the restoration of immune functions in immunocompromised individuals that are subjected to antibiotics and chemotherapy. In a study with **HAB-439** isoxazoline derivative the compound appeared to be an inhibitor of aminopeptidase [33]. **HAB-439** stimulated the DTH response to *Salmonella typhimurium* and *Listeria monocytogenes*. It also restored a decreased DTH reaction that was caused by the ampicillin treatment of mice infected with *L. monocytogenes*.

Several immunostimulatory isoxazole derivatives were synthesized, and their potential therapeutic utility was evaluated by our research team. **RM-11** (3,5-dimethyl-5,6-dihydro-4*H*-[1,2]oxazolo[5,4-*e*][1,2,4]triazepin-4-one) was found to potently stimulate both humoral and cellular immune response to SRBC in mice [34]. The compound also stimulated ConA-induced splenocyte proliferation. **RM-11** showed no sign of toxicity when given to mice at a dose of 250 mg/kg body weight. The compound was subsequently investigated in a model of immunocompromised mice treated with cyclophosphamide (CP) [35]. In this model, the compound, given i.p. in repeatable doses, caused a rapid recovery of both antibody production against SRBC and DTH responses to OVA, in comparison with control mice. In addition, the compound accelerated the process of myelopoiesis as measured by the percentage of neutrophils and their precursors in the peripheral blood. The compound was also protective in the humoral immune response in vitro to SRBS suppressed by methotrexate. The phenotypic studies revealed that **RM-11** preferentially increased the percentage of mature, single positive CD4+ and CD8+ T cells in the spleen of normal mice. Similar results were obtained with another T-cell tropic isoxazole derivative, 3,5-dimethyl-5,8-dihydro-4*H*-[1,2]oxazolo[5,4-*e*][1,2,4]triazepin-4-one hydrochloride (**R 13**), which also induced a significant increase of CD4+ T cells in the spleen, and in the lymph nodes of mice [37]. The compound significantly accelerated both the antibody production and the cellular immune response. Unlike **RM-11**, this compound decreased the content of myelocytic cells in the circulating blood, but increased the level of immature lymphocyte forms, indicating the preferential promotion of lymphopoiesis in CP-treated mice. In contrast to the two previously described compounds, 3,5-dimethyl-5,8-dihydro-4*H*-[1,2]oxazolo[5,4-*e*][1,2,4]triazepin-4-one (**R-11)** appeared to preferentially induce the recruitment of CD19+ B cells in normal mice [36]. Moreover, the development of both the humoral immune response to SRBC and DTH to OVA was also accelerated in CP-immunocompromised mice by this compound. The compound also stimulated IL-6 production, elicited by LPS in human whole blood cultures. We postulate that the above described compounds may be invaluable in the restitution of the immune function of patients undergoing chemotherapy. At present, the only therapeutic that is accepted for this purpose is granulocyte colony stimulating factor (Filgrastim^®^) [55]. However, the use of this cytokine has serious limitations, since it is costly and temperature-sensitive. In addition, this cytokine predominantly promotes myelopoiesis, the process is spontaneously recovered quickly [56]. However, the main problem with the reconstitution of the immune cell compartments of the chemotherapy patients are T- and B-cell compartments, where recovery takes much longer [57].

Other stimulatory izoxazole derivatives included 7-amino-3,5-dimethylisoxazole[5,4-*e*][1,3,4]-triazepin-4-one, showing the stimulatory effect on Con A-induced mouse splenocyte proliferation and cytokine production by the P388D1 macrophage cell line [58] and 2-(5-amino-3-methyl-1,2-oxazole-4-carbonyl)-*N*-(prop-2-en-1-yl)hydrazine-1-carbothioamide (compound **M4**) [12], which stimulated PHA-induced proliferation of human PBMC.

The aim of another study was to determine the immunomodulatory activity of 5-amino-3-methyl-1,2-oxazole-4-carbohydrazide in vitro [38]. The compound was not cytotoxic against reference cell lines, up to a concentration of 200 μg/mL. The compound stimulated the mitogen-induced proliferation of lymphocytes isolated from spleens and mesenteric lymph nodes when they were used alone and in combination with mitogens, and it increased LPS-elicited IL-1β production in peritoneal cell culture.

Interestingly, among salicylic acid derivatives containing an isoxazole ring, 3-(4-methoxyphenyl)-4-(3-hydroxy-4-carboxybenzoyl)-5-(3-chlorophenyl)-4,5-dihydroisoxazoline (**8e**), which demonstrated mitogenic activity towards human lymphocytes and mouse splenocytes, was found [39]. The ability of the compound to stimulate cell division was caused by increased IL-2 secretion. It seems that an advantage of this new mitogenic compound is that it should not bind to and inactivate sugar-containing biologically active proteins, in contrast to lectins (ConA, PHA). A property of binding to sugars in biologically active glycoproteins like lactoferrin [59] by protein mitogens (lectins) may hamper the interpretation of results deriving from models where mitogenic lectins are applied.

## 3. Conclusions

The isoxazole derivatives described herein have differential activities and mechanisms of action, and it may be applied in such disease and immunological disorders as inflammation, infection, autoimmune disorders, and impaired immune responsiveness. It is worthy to underline that many of the compounds, presented in this review, have higher potency in comparison to relevant reference drugs; thus they are potential therapeutics and they may replace registered drugs. Alternatively, they may enrich the pool of existing drugs in the market. Keeping in mind that even registered drugs are not completely devoid of side-effects and toxicity, the search for new compounds with the possible highest selectivity of action and the lowest toxicity is highly justified.

## Figures and Tables

**Table 1:** Isoxazole derivatives with potential application in therapy.

Compound	Structure	Activity	Molecular/Cellular Mechanism of Action	Reference Number
**Immunosuppressive actions**				
**HWA-486**/Leflunomide 5-methyl-*N*-[4-(trifluoromethyl)phenyl]-1,2-oxazole-4-carboxamide	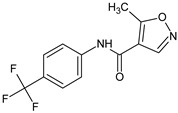	Immunosuppressive and anti-inflammatory regulation of autoimmune lymphocytes	COX-2 inhibitor, non-cytotoxic inhibitor of dihydroorotate dehydrogenase	[6]
Parecoxib	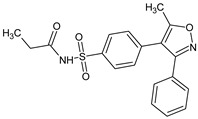	Anti-inflammatory registered drug	COX-2 inhibitor	[7]
**8g**/5-(4-amino-5-benzoyl-1,2-oxazol-3-yl)-*N*-[(pyridin-4-yl)methyl]-1,3,4-oxadiazole-2-carboxamide	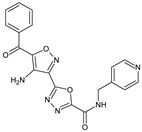	Inhibits the proliferative response of mouse splenocytes to concanavalin A, suppresses the humoral immune response	N.D.	[8]
**10f**/5-(5-amino-7-phenyl[1,2]oxazolo[4,5-d]pyrimidin-3-yl)-*N*-[(pyridin-3-yl)methyl]-1,3,4-oxadiazole-2-carboxamide	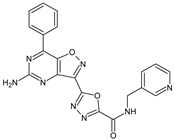	Stimulates mitogen-induced proliferation of mouse splenocytes, suppresses DTH	N.D.	[8]
**4d**/5-(2-hydroxyethyl)piperazinomethinimino-3-methyl-4-isoxazolecarboxylic acid 4-(4-ethoxyphenyl)-amide	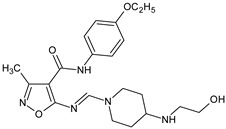	Inhibits the DTH and humoral immune response to SRBC in vitro and in vivo	N.D.	[9]
**RM-33**/3,5,7-trimethyl-5,6,7,8-tetrahydro-4*H*-[1,2]oxazolo[5,4-*e*][1,2,4]triazepin-4-one	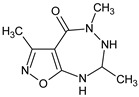	Inhibits LPS-induced TNF-α and IL-6 activity inhibits antibody production	* Stimulation of caspase 9 expression in thymocytes and splenocytes and Fas in bone marrow cells and splenocytes, inhibition of ERK1 and p38g in bone marrow cells	[10]
**1020**/5-{[(4-hydroxyphenyl)methylidene]amino}-3-methyl[1,2]oxazolo[5,4-*d*]pyrimidin-4(5*H*)-one	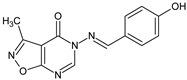	Displays strong immunosuppressive action, inhibits polyclonal antibody production	N.D.	[11]
**1025**/5-(cyclohexylideneamino)-3-methyl[1,2]oxazolo[5,4-*d*]pyrimidin-4(5H)-one	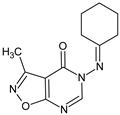	Shows immunosuppressive property, lowers polyclonal antibody production	N.D.	[11]
**M5**/2-[(5-amino-3-methylisoxazol-4-yl)carbonyl]-*N*-(4-chlorophenyl)hydrazinecarboxamide	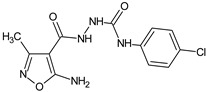	Inhibits antibody production in mice	N.D.	[12]
**RM56**/5-(5-amino-3-methyl-1,2-oxazol-4-yl)-1,3,4-oxadiazol-2-amine	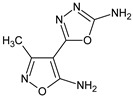	Inhibits the humoral immune response, the carrageenan reaction and proliferation of lymphocytes	N.D.	[13]
**MO5**/5-amino-3-methyl-*N*-[(4-methylphenyl)methyl]-1,2-oxazole-4-carboxamide	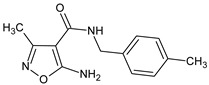	Inhibits the humoral immune response in vitro	Inhibitor of TNFα production	[14]
5-amino-3-methyl-1,2-oxazole-4-carbohydrazide	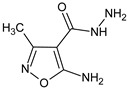	Modulates the content of T cell subsets and B cells in lymphoid organs, and elevates the humoral immune response in mice	Upregulation of fractalkine (CX3CL1) and IL-17F, and downregulation of IL-10 and TLR4	[15]
**MM3**/5-amino-*N*′-[2-(2,4-dihydroxyphenyl)ethylidene]-*N*,3-dimethyl-1,2-oxazole-4-carbohydrazide	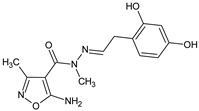	Inhibits the mitogen-induced proliferation of PBMC and LPS-induced TNF α production in human blood cell culture	Strong increases in the expression of caspases, Fas, and NF-kB1	[16]
**Immune regulators**				
**HWA-486**/Leflunomide 5-methyl-*N*-[4-(trifluoromethyl)phenyl]-1,2-oxazole-4-carboxamide	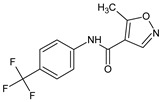	Suppresses T cell-dependent B-cell responses, does not affect T-independent B-cell function	COX-2 inhibitor	[17]
5-amino-3-methyl-1,2-oxazole-4-carbohydrazide	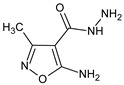	Modulates T cell subset composition and the levels of B cells in lymphoid organs, and enhances anti-SRBC humoral immune responses in mice	Modulation of T and B cell content in lymphatic organs	[18]
**06K**/2-(5-amino-3-methyl-1,2-oxazole-4-carbonyl)-*N*-(4-chlorophenyl)hydrazine-1-carbothioamide	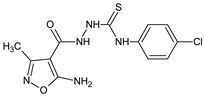	Induces lymphopoiesis and the generation of regulatory T-cells, stimulates the humoral immune response, decreases the DTH reaction	Increases percentage of CD8+ and regulatory CD4+CD25+Fox3+ T cells in spleens and lymph nodes	[19]
**01K**/2-(5-amino-3-methyl-1,2-oxazole-4-carbonyl)-*N*-phenylhydrazine-1-carbothioamide	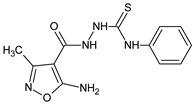	Regulates proliferation of thymocytes, splenocytes, and lymph node cells	Regulation of IL-1β and TNF-α production in peritoneal cell cultures	[20]
**MO5**/5-amino-3-methyl-*N*-[(4-methylphenyl)methyl]-1,2-oxazole-4-carboxamide	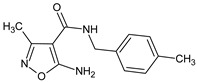	Inhibits the humoral immune response in vitro	Inhibition of TNFα production	[14]
**Anti-inflammatory**				
5-amino-*N*-(4-ethoxyphenyl)-3-methyl-1,2-oxazole-4-carboxamide	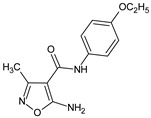	Lowers carrageenan-induced paw edema, exhibits antibacterial activity	N.D.	[21]
5-benzamido-*N*-(4-chlorophenyl)-3-methyl-1,2-oxazole-4-carboxamide	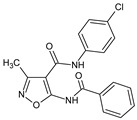	Shows anti-inflammatory activity in the carrageenan-induced reaction, has antibacterial properties	N.D.	[21]
**VGX-1027** (S,R)-(3-phenyl-4,5-dihydro-1,2-oxazol-5-yl)acetic acid	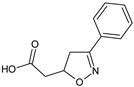	Suppresses carrageenan-induced pleurisy, LPS-induced lethality, and type II-collagen-induced arthritis	Suppression of TNF α, IL-1β, MIF, inhibition of NFκB and p38, and upregulation of ERK signaling	[22]
*N*-(2-(Benzo[*d*][1,3]dioxol-5-yl)ethyl)-5-(biphen-4-yl)-isoxazole-3-carboxamide	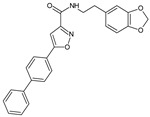	Protects against experimental colitis	Inhibition of FAAH (fatty acid amide hydrolase))	[23]
**2b**/5-(3-methylthiophen-2-yl)-3-(3,4,5-trimethoxyphenyl)-1,2-oxazole	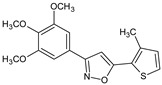	Inhibits tumor growth, peritoneal angiogenesis, and ascite formation in Erlich carcinoma mouse model	Inhibitory activity toward lipooxygenase (LOX) and COX-2	[24]
**39**/4-(4-Chlorophenyl)-5-[4-(quinolin-2-ylmethoxy)phenyl]isoxazol-3-carboxylic acid **40**/4-(4-Chlorophenyl)-5-[4-(benzothiazol-2-ylmethoxy)phenyl] isoxazol-3-carboxylic acid	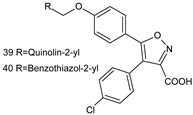	Anti-inflammatory agent	Inhibitory activity against cellular 5-LO (5-lipoxygenase) product synthesis	[25]
**ISO-1**/(S,R)-methyl [3-(4-oxocyclohexa-2,5-dien-1-ylidene)-1,2-oxazolidin-5-yl]acetate	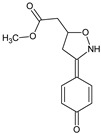	Blocks corticosteroid-insensitive lung inflammation and airway hyper-responsiveness) suppresses lung inflammation in ozone-exposed mice, in contrast to dexamethasone	Inhibition of migration inhibitory factor (MIF) function	[26]
**Cpd #15**/(16S,17R)-30-bromo-6,9-difluoro-11b,21-dihydroxy-40*H*-pregna-1,4-dieno[16,17-*d*]isoxazole-3,20-dione	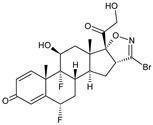	Inhibits eosinophilic infiltration in a model of allergen-induced pulmonary inflammation in rats	Inhibits LPS-induced nitric oxide production	[27]
**7a**/*N*-(4-(isoxazol-5-yl)phenyl)-4,6-di(piperidin-1-yl)-1,3,5-triazin-2-amine	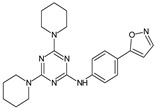	Reduces carrageenan-induced mice paw edema in comparison to celecoxib	COX-2 inhibitor	[28]
**MZO-2**/ethyl *N*-(4-{[(2,4-dimethoxyphenyl)methyl]carbamoyl}-3-methyl-1,2-oxazol-5-yl)ethanimidate	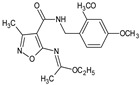	Inhibits carrageenan-induced footpad inflammation and the contact sensitivity in mice to oxazolone when applied in ointment	Suppression of LPS-induced TNFα, and the expression of caspases 3, 8, and 9 in Jurkat cells	[29]
4-(2-(4-(1*H*-benzimidazol-2-yl)phenyl)hydrazono)-1-(4-chlorophenyl)-3-methyl-1*H*-pyrazol-5(4*H*)-one	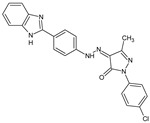	Demonstrates analgesic activity and protection in paw edema test, is comparable with that of the reference drug Diclofenac	N.D.	[30]
Isoxazole-mercaptobenzimidazole hybrids	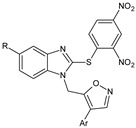	Is analgesic and anti-inflammatory activity in vivo	N.D.	[31]
(3S,3aR,6aS)-5-benzyl-3-(1H-indol-3-yl)-2-phenyl-hexahydro-2H-pyrrolo[3,4-*d*][1,2]oxazole-4,6-dione (**9ª**)	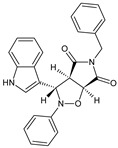	Increases survival in a mouse model of sepsis	Inhibition of LPS-induced TNF α and IL-6 production in macrophage THP-1 cells	[32]
**Immunostimulatory**				
**HAB-439**/(3-phenyl-4,5-dihydro-1,2-oxazol-5-yl)phosphonic acid	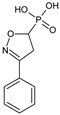	Stimulates DTH response to *Salmonella typhimurium* and *Listeria monocytogenes*	Inhibitor of aminopeptidase B	[33]
**RM-11**/3,5-dimethyl-5,6-dihydro-4*H*-[1,2]oxazolo[5,4-*e*][1,2,4]triazepin-4-one	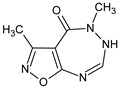	Stimulates mitogen-induced splenocyte proliferation and accelerates the restoration of the humoral and cellular immune response in cyclophosphamide-treated mice	N.D.	[34,35]
**R-11**/3,5-dimethyl-5,8-dihydro-4*H*-[1,2]oxazolo[5,4-*e*][1,2,4]triazepin-4-one	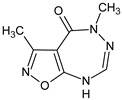 (base)	Recruits CD19+ B and accelerates the development of both types of the immune response in immunocompromised mice	Stimulation of LPS-induced IL-6 production in human whole blood cultures	[36]
**R-13**/3,5-dimethyl-5,8-dihydro-4*H*-[1,2]oxazolo[5,4-*e*][1,2,4]triazepin-4-one hydrochloride	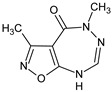 + HCL (salt)	Recruits CD4+ T cells and accelerates the restoration of both types of the immune response in immunocompromised mice	N.D.	[37]
**M4**/.2-(5-amino-3-methyl-1,2-oxazole-4-carbonyl)-*N*-(prop-2-en-1-yl)hydrazine-1-carbothioamide	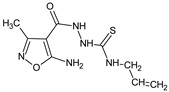	Enhances the mitogen-induced proliferative response of human PBMC	N.D.	[12]
5-amino-3-methyl-1,2-oxazole-4-carbohydrazide	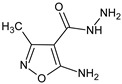	Stimulates mitogen-induced proliferation of splenocytes and lymph node cells	Increase of LPS-induced induced IL-1β production by peritoneal cells	[38]
**8e**/3-(4-methoxyphenyl)-4(3-hydroxy-4-carboxybenzoyl)-5-(3-chlorophenyl)-4,5-dihydrodihydroisoxazoline	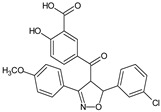	Augments the proliferative response of human and mouse lymphocytes	Increased IL-2 secretion	[39]

* Recently established, unpublished; N.D.—not defined.
